# Traditional prenatal and postpartum food restrictions among women in northern Lao PDR

**DOI:** 10.1111/mcn.13273

**Published:** 2021-09-30

**Authors:** Taryn J. Smith, Xiuping Tan, Charles D. Arnold, Dalaphone Sitthideth, Sengchanh Kounnavong, Sonja Y. Hess

**Affiliations:** ^1^ Institute for Global Nutrition University of California Davis Davis California USA; ^2^ Lao Tropical and Public Health Institute Vientiane Lao People's Democratic Republic

**Keywords:** diet, dietary diversity, food taboos, Lao PDR, maternal nutrition, postpartum, pregnancy

## Abstract

Culturally determined food restrictions are common among pregnant and postpartum women in Asia. This study aimed to describe perinatal dietary restrictions, factors associated with food avoidances and attainment of minimum dietary diversity (MDD‐W) among women in Lao PDR. Mother–child (aged 21 days to <18 months) dyads (*n* = 682) were enrolled into a cohort study in northern Lao PDR and interviewed at one time point postpartum. During pregnancy and postpartum, 1.6% and 97% of women reported following dietary restrictions, respectively. Cluster analysis identified four distinct postpartum dietary patterns: most restrictive (throughout first 2 months postpartum); least restrictive; 2 weeks highly restrictive and 1 month highly restrictive, followed by 19%, 15%, 5% and 62% of women, respectively. Greater maternal age, gravidity and higher household socioeconomic status were associated with allowing more diverse foods, while women from food insecure households followed more restrictive diets for longer. Women belonging to the Hmong ethnic group followed a highly restrictive diet of white rice and chicken for the first month postpartum. MDD‐W was achieved by 10% of women restricting their diet at the time of the interview compared with 17% of women who were consuming their normal diet (*p* = 0.04). Postpartum dietary restrictions are widespread among women in northern Lao PDR. These highly restrictive diets, low dietary diversity and food insecurity likely contribute to micronutrient deficiencies in women that may have important consequences for their breastfed infants through reduced breastmilk micronutrient content, which requires further exploration. Culturally appropriate strategies to increase micronutrient intakes among women should be considered.

**Key messages**

- Traditional highly restrictive diets are common among postpartum women in northern Lao PDR, in contrast to pregnancy food avoidances which were not commonly practiced.
- Food insecurity was associated with longer and more restrictive postpartum diets. Greater maternal age, gravidity and household socioeconomic status were associated with allowing more diverse foods and shorter duration of dietary restrictions.
- Dietary diversity was low among the majority of women and exacerbated by culturally determined postpartum food avoidances.
- Highly restrictive diets and low dietary diversity likely contribute to micronutrient deficiencies in women that may have important consequences for their breastfed infants, which requires further exploration.

## 1 INTRODUCTION

Adequate nutrition during pregnancy and lactation is critical to support maternal health and foetal and infant growth and development. This is especially important in resource poor settings where low dietary diversity and inadequate intakes, in combination with increased nutrient requirements, place pregnant and lactating women at high risk of nutritional deficiencies.

Like many countries in the region, there is a high burden of malnutrition among women and children in Lao People's Democratic Republic (Lao PDR), a predominantly rural and ethnically diverse lower–middle income country in Southeast Asia. The most recent Lao Social Indicator Survey (LSIS II) in 2017 documented high levels of stunting (33%), underweight (21%) and wasting (9%) among children less than 5 years of age (Lao Statistics Bureau, 2018). Although rates have been declining over recent years, malnutrition remains particularly prevalent in the rural northern and southern provinces, which are largely populated by minority ethnolinguistic groups, and is attributable to higher levels of poverty and limited access to healthcare services, infrastructure, sanitation and maternal education (Osornprasop & Pimhidzai, 2016). Although data on the micronutrient status of women and children in Lao PDR is limited, anaemia is a concern, with a prevalence of 40% among women aged 15–49 years and 44% among children aged 6–59 months (Lao Statistics Bureau, 2018). Recent studies have also reported zinc (Barffour et al., 2019) and thiamine deficiencies (Barennes et al., 2015; Khounnorath et al., 2011; Soukaloun et al., 2003) among infants and young children.

Rice is the predominant dietary staple in Lao PDR, consumed as either glutinous sticky rice or white rice, often eaten with small quantities of meat, poultry or fish and vegetables (World Food Programme, 2017). While food insecurity is assumed to be a major determinant of malnutrition in Lao PDR, analysis by The World Bank demonstrated that insufficiently diverse diets are of greater concern than meeting caloric intake (Osornprasop & Pimhidzai, 2016). In a nationally representative food consumption survey, >80% of pregnant and lactating women were estimated to have insufficient intakes of vitamin A, thiamine, riboflavin, niacin and calcium (Ratsavong et al., 2020).

Culturally determined perinatal dietary restrictions and food avoidances likely exacerbate inadequate dietary micronutrient intakes. Food taboos have been defined as a deliberate avoidance of foods for reasons other than simple dislike from food preferences, often passed through generations as a cultural practice in connection with various milestones, such as birth (Meyer‐Rochow, 2009). Food taboos can be found in societies globally, although foods considered as taboo and the reasons attached to the taboos vary between societies (Kavle & Landry, 2018). Perinatal food avoidances are commonly practised across South and Southeast Asia (Köhler et al., 2018, 2019), including Lao PDR where it has previously been reported that 80–98% of mothers adhered to postpartum food restrictions (Barennes et al., 2009, 2015; de Sa et al., 2013). Restrictive diets during pregnancy are less common, but the practice of ‘eating down’ to avoid a perceived difficult delivery of a large baby has been reported in Lao PDR (Holmes et al., 2007).

Although local knowledge of restrictive diets in the perinatal period is widespread in Lao PDR, there has been little in‐depth exploration of these practices during pregnancy and postpartum or the underlying sociocultural reasons and beliefs. Therefore, the objectives of this study are to (1) describe the traditional food restrictions during pregnancy and postpartum by women in northern Lao PDR and underlying reasons; (2) explore factors associated with adherence to food restrictions and (3) determine if there are differences in the minimum dietary diversity for women adhering to a traditional restricted diet at the time of the interview compared with women not following a restricted diet.

## 2 METHODS

### 2.1 Study design, setting and population

This is a secondary analysis of data collected from mother–child pairs who participated in a hospital‐ and community‐based prospective cohort study in Luang Prabang, northern Lao PDR. The primary aim of the study was to develop a case definition for thiamine responsive disorders among infants and young children, for which the study protocol has previously been described in detail (Hess et al., 2020). Briefly, infants and young children aged 21 days to <18 months seeking care at the Lao Friends Hospital for Children in Luang Prabang, with symptoms consistent with thiamine deficiency disorders, were eligible for participation. A community cohort of infants and young children frequency‐matched by age, sex and village of residence were enrolled to serve as a comparison group. On a weekly basis, characteristics of hospitalized children were summarized, and community children were enrolled with the assistance of health centres and village heads, who established contact with the child's parents/caregivers and invited them for a study appointment at the health centre. Mothers of hospitalized and community children were also invited for participation in the study providing informed consent was obtained.

### 2.2 Data collection

A comprehensive questionnaire was developed to interview women about their diets during pregnancy and postpartum. In Lao PDR, restrictive diets during the perinatal period are locally referred to as ‘taboo diets’, and it was therefore contextually appropriate to use the term ‘taboo diet’ when interviewing mothers. Specifically, women were asked what foods they allowed and which were restricted during each trimester of pregnancy, the period shortly before birth and in weekly (for the first 4 weeks) and monthly intervals postpartum until 6 months, and then subsequent months grouped together. Women who followed a taboo diet during pregnancy were asked when they started the taboo diet (first, second or third trimester or shortly before birth) and if her taboo diet changed over the course of her pregnancy. Women were interviewed up until the time period consistent with the child's age. Questions began at the food group level (e.g., grains, vegetables and poultry) and then advanced to determining if all foods or specific food items within food groups were allowed/restricted (e.g., sticky rice, cabbage and chicken). If the woman had resumed eating her habitual diet at the time of the interview, it was determined at what age the child was when she finished her taboo diet. Conversely, if the woman was following a restricted diet at the time of the interview, she was asked how long she planned to follow the taboo diet for. Reasons for restricting foods during pregnancy and postpartum were also recorded, as well as reasons for the woman returning to her normal diet.

To determine the mother's dietary diversity, women were asked to recall foods consumed in the previous 24 h. As mothers of hospitalized children may have changed their dietary intake due to their presence at the hospital, these women were asked about their dietary intake for the day prior to their arrival at the hospital. First, women were asked to openly recall and list all foods consumed in the previous 24 h/day before going to the hospital, and then a list‐based method was used to ask the mother if she had consumed any foods in the previous 24 h/day before going to the hospital from pre‐defined food groups (FAO & FHI 360, 2016). Consumption of ≥5 out of 10 defined food groups in the previous 24 h/day before going to hospital was considered as meeting the minimum dietary diversity for women (MDD‐W). Caregiver recall of infant feeding practices using structured survey questions was used to determine breastfeeding status (World Health Organization, 2010).

Self‐reported indicators of socioeconomic status (SES) included education and occupation of the mother and household head, household size and composition, housing characteristics, access to utilities and household ownership of assets and land. These proxy indicators were used to estimate household SES index using principal component analyses (Vyas & Kumaranayake, 2006). Food security was assessed using the Household Food Insecurity Access Scale (Coates et al., 2007). Maternal social desirability, defined as the tendency of respondents to answer questions in a manner that is viewed favourably by others, was assessed to evaluate the accuracy of self‐reported measures using five brief questions as previously proposed (Menon et al., 2016; Reynolds, 1982). A social desirability score was created by adding up the number of socially desirable responses (0–2 = low score; 3 = medium score; 4 = high score; 5 = very high score; Menon et al., 2016). Mothers were also interviewed about the child's birth history and the child's and mother's health history.

Maternal height, weight and left mid‐upper arm circumference (MUAC); child recumbent length and weight and maternal and child complete blood count using venous blood were assessed following standard protocols as previously described (Hess et al., 2020).

All data were entred electronically into Samsung tablets (Samsung Galaxy Tab 3V, Seoul, South Korea) with the use of SurveyCTO (Dobility, Cambridge, Massachusetts, USA).

### 2.3 Ethical considerations

Ethical approval was obtained from the National Ethics Committee for Health Research, Ministry of Health, Lao PDR, and the Institutional Review Board of the University of California Davis. Written or finger‐printed informed consent was obtained from at least one primary caregiver for the child's participation in the study, and from the mother for her own participation, after a detailed explanation of the study in a language appropriate to the family.

### 2.4 Statistical analysis

A detailed statistical analysis plan was developed and published prior to analysis (Hess et al., 2019). Descriptive statistics are presented as mean ± standard deviation for continuous variables and frequencies (percentages) for categorical variables. Independent *t* tests for continuous data and Pearson's chi‐squared test for categorical data were used to compare differences between groups.

Food items reported from the taboo diet questionnaire were consolidated into 12 food groups: grains, vegetables, fruits, meat, poultry, fish, wild animals, roots and tubers, pulses, nuts and seeds, eggs and dairy (FAO & FHI 360, 2016). Each food group was defined as a dichotomous variable indicating whether the woman allowed or restricted two or more food items within the food group at any given prenatal and postpartum time point, based on assumptions previously outlined in the statistical analysis plan (Hess et al., 2019). To identify women with similar dietary patterns, clustering analysis was performed using a *k*‐means matching method (Sokal & Michener, 1958). To determine the optimal number of clusters, a series of cluster solutions were analysed with the within‐cluster sum of squares (WSS) based on the simple matching distance. The final number of clusters was chosen based on the reduction of WSS and the interpretability of the cluster size. Each cluster derived represents a dietary pattern group.

Multivariable logistic regression analyses were conducted to assess the relationship between dietary patterns and maternal and household socio‐demographic characteristics as predictor variables while controlling for whether women were interviewed at the hospital or in the community. First, potential factors associated with the diet pattern and food groups were evaluated using minimally adjusted bivariate logistic regression models. All predictors associated with the outcome at a level of *p* value <0.1 were then included in the multivariable model. Multicollinearity was assessed using the variance inflation factors. Results are presented as odds ratio (OR) and 95% confidence intervals (CI). All tests were two sided, at a 5% level of significance. All statistical analyses were performed using Stata (version 14.2) and R (version 3.6.2).

## 3 RESULTS

### 3.1 Participants' characteristics

A total of 782 women and their children were identified as potentially eligible for participation in the study, of which 699 were enrolled (Figure S1). Considering the focus on taboo diets in the present analyses, women‐child pairs were excluded if the primary female caregiver was not the biological mother (*n* = 9) or where the taboo diet questionnaire had not been completed (*n* = 8), resulting in 682 mother–child pairs included in the analysis.

Characteristics of women and their children are shown in Table 1. Mean ± SD age of women and children was 24.7 ± 6.3 years and 4.3 ± 3.3 months, respectively, and 95% of mothers were breastfeeding their child. Almost half of all women (48%) belonged to the Hmong ethnic group, followed by Khmu (33%) and Lao (15%) ethnic groups; however, this differed among the hospital and community cohorts. Moderate and severe household food insecurity was higher among the hospital cohort compared with the community cohort (40% and 24%, respectively; *p* < 0.001), and the SES index was lower in the hospital cohort (*p* < 0.001). Mean ± SD gravidity was 2.6 ± 1.8, 8% of women were underweight and 42% had a MUAC <23.5 cm, indicating maternal malnutrition, and 26% were anaemic; the prevalence of which was greater among the hospital compared with the community cohort (31% vs. 17%, respectively; *p* < 0.001). Among children, 25% were stunted, 8% were wasted, 21% were underweight and 44% were anaemic; the prevalence of all indicators of malnutrition was greater among the hospital cohort (all *p* < 0.001).

**Table 1:** Characteristics of women and their children in the hospital and community cohorts

Characteristics	All (*n* = 682)	Hospital (*n* = 433)	Community (*n* = 249)	*p* value
Women				
Age (years)	24.7 ± 6.3	24.6 ± 6.4	24.8 ± 6.2	0.77
Province of residence				0.23
Luang Prabang	622 (91)	396 (91)	226 (91)	
Oudomxay	39 (6)	21 (5)	18 (7)	
Xayaboury	12 (2)	7 (2)	5 (2)	
Other	9 (1)	9 (2)	0 (0)	
Ethnic group				<0.001
Lao	104 (15)	49 (11)	55 (22)	
Khmu	224 (33)	104 (24)	120 (48)	
Hmong	329 (48)	269 (62)	60 (24)	
Other	25 (4)	11 (3)	14 (6)	
Occupation				<0.001
Does not work	9 (1)	4 (1)	5 (2)	
Housewife	142 (21)	75 (17)	67 (27)	
Farmer	431 (63)	308 (71)	123 (49)	
Unskilled labourer	4 (1)	3 (1)	1 (1)	
Skilled worker	96 (14)	43 (10)	53 (21)	
Education				<0.001
No formal education	132 (19)	102 (24)	30 (12)	
Some/completed primary	211 (31)	125 (29)	86 (35)	
Some/completed secondary	294 (43)	186 (43)	108 (43)	
College/university	45 (7)	20 (5)	25 (10)	
Gravidity	2.6 ± 1.8	2.7 ± 2.0	2.3 ± 1.5	0.005
BMI (kg/m^2^)^a^				0.49
<18.5	57 (8)	38 (9)	19 (8)	
≥18.5–24.9	521 (78)	332 (79)	189 (76)	
≥25.0–29.9	86 (13)	48 (11)	38 (15)	
≥30.0	6 (1)	4 (1)	2 (1)	
Height <150 cm^a^	362 (54)	253 (60)	109 (44)	<0.001
MUAC <23.5 cm^b^	286 (42)	190 (45)	96 (39)	0.14
Anemia^c^	175 (26)	132 (31)	43 (17)	<0.001
Household SES index^d^	0.0 ± 1.0	−0.1 ± 1.0	0.2 ± 1.0	<0.001
Food insecurity category^e^				<0.001
Moderate to severe	230 (34)	171 (40)	59 (24)	
Mild to none	451 (66)	261 (60)	190 (76)	
Dietary diversity score	3.2 ± 1.4	3.0 ± 1.4	3.5 ± 1.3	< 0.001
Achieved MDD‐W	100 (15)	54 (13%)	46 (19%)	0.03
Children				
Age (months)	4.3 ± 3.3	4.3 ± 3.4	4.4 ± 3.2	0.46
Male	406 (60)	262 (61)	144 (58)	0.49
Breastfeeding status^f^				<0.001
Exclusive breastfeeding	421 (62)	272 (63)	149 (60)	
Predominant breastfeeding	45 (6)	37 (9)	8 (3)	
Partial breastfeeding	42 (6)	31 (7)	11 (4)	
Continued breastfeeding	141 (21)	66 (15)	75 (30)	
No longer breastfeeding	33 (5)	27 (6)	6 (2)	
Stunted^g^	167 (25)	131 (32)	36 (15)	<0.001
Wasted^g^	52 (8)	47 (12)	5 (2)	<0.001
Underweight^g^	135 (21)	114 (28)	21 (8)	<0.001
Anemia^h^	285 (44)	224 (55)	61 (26)	<0.001

*Note:* Values represent mean ± SD for continuous variables or n (%) for categorical variables.

Abbreviations: BMI, body mass index; MDD‐W, minimum dietary diversity for women; MUAC, mid‐upper arm circumference; SES, socioeconomic status.

^a^ *n* = 670; *n* = 422 hospital; *n* = 248 community.

^b^ *n* = 674; *n* = 426 hospital; *n* = 248 community.

^c^ Haemoglobin cut‐offs: <120 g/L for non‐pregnant women; <110 g/L for pregnant women; *n* = 677; *n* = 429 hospital; *n* = 248 community.

^d^ SES index derived from self‐reported measures of housing characteristics, household access to utilities and household ownership of assets and land; *n* = 678; *n* = 431 hospital; *n* = 247 community.

^e^ Food insecurity assessed using the Household Food Insecurity Access Scale (Coates et al., 2007); *n* = 681; *n* = 432 hospital; *n* = 249 community.

^f^ Predominant breastfeeding defined as breastfeeding with certain liquids (water, water‐based drinks, fruit juice); partial breastfeeding defined as breastfeeding with other beverages, milk from cows or buffalo, infant formula.

^g^ *n* = 657; n = 408 hospital; *n* = 249 community.

^h^ Haemoglobin cut‐offs: <110 g/L for children; *n* = 650; n = 411 hospital; *n* = 239 community.

### 3.2 Dietary restrictions during pregnancy

Eleven (1.6%; *n* = 10 in the hospital cohort and *n* = 1 in the community cohort; *p* = 0.06) women reported following a taboo diet during pregnancy, of which eight women reported starting the taboo diet during the first trimester, while three women started in the second trimester. Only one woman reported restricting all or most foods during pregnancy and only allowed rice, chicken and vegetables. Other food groups that were restricted and the reasons for restricting these foods during pregnancy are shown in Table 2. Tradition within the family/ethnic group was the main reason given for restricting foods. Half of women reported that they ate more during pregnancy, 39% reported eating the same and 11% reported eating less.

**Table 2:** Reasons for restricting all or most or specific food groups during pregnancy and postpartum

Reasons	All or most foods^a^	Meat	Poultry	Fish	Wild animals	Frogs and snakes	Insects	Vegetables	Fruits	Roots and tubers	Other foods^b^
Pregnancy											
Avoid having a large baby	0	0	0	0	1	0	0	0	0	0	0
Tradition in family/ethnic group	1	5	1	2	1	1	2	1	5	4	3
Cause pain in the women's body	0	0	0	1	0	0	0	0	0	0	0
Postpartum											
Tradition in family/ethnic group	471	92	7	21	27	13	5	1	2	0	43
Help the mother's body heal after childbirth	169	2	1	4	0	0	0	1	0	0	10
Cause pain in the mother's body	19	1	0	2	0	0	0	1	0	0	5
Cause pain in the infant	8	0	0	0	0	0	0	0	0	0	0
Lead to illness in the infant	1	0	0	0	0	0	0	0	0	0	0
Improve breastmilk quality and/or quantity	0	0	0	0	0	0	0	0	0	0	0

*Note:* Values represent number of responses (not number of women).

^a^ Multiple reasons could be given for restricting all or most foods.

^b^ Other foods specified by women: pickled foods (*n* = 2) and cake (*n* = 1) during pregnancy; pickled foods (*n* = 58) postpartum.

### 3.3 Postpartum dietary restrictions

Almost all women (97%; *n* = 426 [98%] and *n* = 235 [94%] of the hospital and community cohorts, respectively; *p* = 0.004) reported following a taboo diet postpartum for a median (IQR) length of time of 1 (1, 3) month, but ranging from 1 week to 36 months. Sixty‐five percent of women reported adhering to food restrictions for ≤1 month postpartum, 35% for ≥2 months, 25% for ≥3 months, 20% for ≥4 months, 16% for ≥5 months and 12% for >6 months. The main reason cited for restricting all or most foods or specific food groups postpartum was tradition within the family/ethnic group, followed by helping the mother's body heal after childbirth (Table 2). All women reported that the reason for resuming their normal diet was because it was the right time to finish the taboo diet.

**Figure 1:**
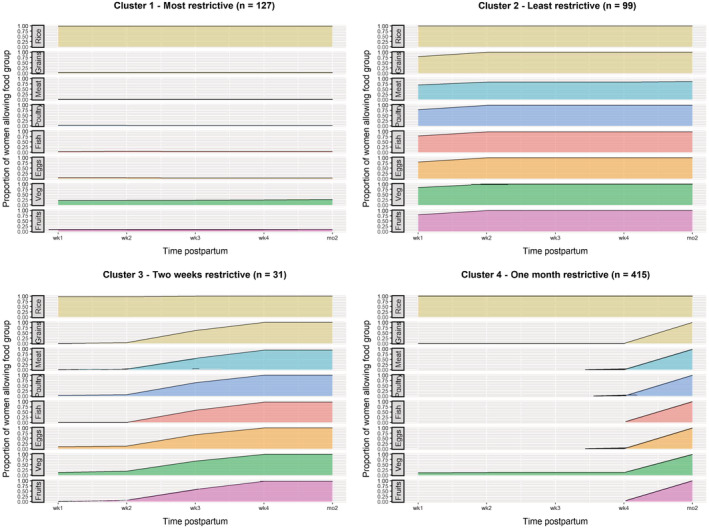
Proportion of women within each cluster who reported allowing food groups during the first 2 months postpartum

Four dietary pattern groups were identified using cluster analysis based on 672 women with complete months 1 and 2 taboo diet data (Figure 1). All women allowed rice at all postpartum time points.

The first cluster of women had the most restrictive diets throughout the first 2 months postpartum (*n* = 127; 19%). Of women in this cluster, in months 1 and 2 postpartum, 21–26% reported allowing vegetables, 8–10% allowed fruits and ≤5% allowed other grains, fish, poultry, wild animals and eggs (Figure 1; Table S1). No women allowed meat in the first 2 months postpartum. Sixty‐nine per cent of women in this cluster followed a taboo diet for >2 months postpartum.

The second cluster of women followed the least restrictive taboo diets (*n* = 99; 15%). Of women in this cluster, 16–20% reported restricting fruits, vegetables, other grains and eggs in week 1 postpartum, but there were no further restrictions from the second postpartum week onwards. Twenty‐one and 22% reported restricting fish and poultry in week 1, respectively, but ≤2% restricted these foods thereafter. For other animal source foods, 30%, 16% and 13% of women restricted meat in week 1, weeks 2–4 and month 2 postpartum, respectively, while 28%, 8% and 6% restricted wild animals in postpartum week 1, weeks 2–4 and month 2, respectively. These women generally allowed most foods from postpartum week 2 onwards, with some restrictions on animal source foods into the second month postpartum.

A third cluster of women reported following a highly restrictive diet for the first 2 weeks postpartum (*n* = 31; 5%), with ≤6% allowing other grains, fruits, fish, poultry, meat and wild animals. In weeks 1 and 2 postpartum, 13% and 19%, respectively, allowed vegetables and 10% and 13%, respectively, allowed eggs. However, by the third week postpartum, women reported gradually allowing more diverse foods, with 52–68% allowing different food groups. By week 4 postpartum, women had largely resumed their normal diets, except for some restrictions which continued into month 2 (10% of women restricted wild animals, 6% meat, 3% fish and 3% fruits).

Finally, a fourth cluster of women reported following a highly restrictive diet for 1 month postpartum and resumed their normal diet in the second month (*n* = 415; 62%). Of women in this cluster, in the first month postpartum, 12–13% reported allowing vegetables, 2% fish and 1% eggs. No women allowed poultry, meat, wild animals or other grains for 1 month. Women had resumed their normal diet in the second month postpartum with no dietary restrictions, with the exception of 2% of women who restricted meat and wild animals.

The proportion of women from each ethnic group in the four clusters is shown in Table S2. Eighty‐five per cent of women of Hmong ethnicity were grouped into the 1 month restrictive cluster. In contrast, women of Khmu ethnicity were more evenly grouped into the 1 month restrictive cluster (38%), the most restrictive (30%) and the least restrictive clusters (25%). Similarly, 43% of Lao women were grouped into the 1 month restrictive cluster, with 31% and 20% into the least and most restrictive clusters, respectively. Foods were restricted for longer among Khmu women (Figure S2). For example, 37%, 29% and 20% of Khmu women reportedly restricted meat in months 2, 3 and 4 postpartum, respectively, compared with 27%, 15% and 13% of Lao women and 11%, 4% and 4% of Hmong women. Similar patterns were seen for all other food groups (Figure S2). From postpartum month 5 onwards, the proportion of women within each ethnic group who restricted each food group was similar.

### 3.4 Factors associated with postpartum dietary restrictions

**Figure 2:**
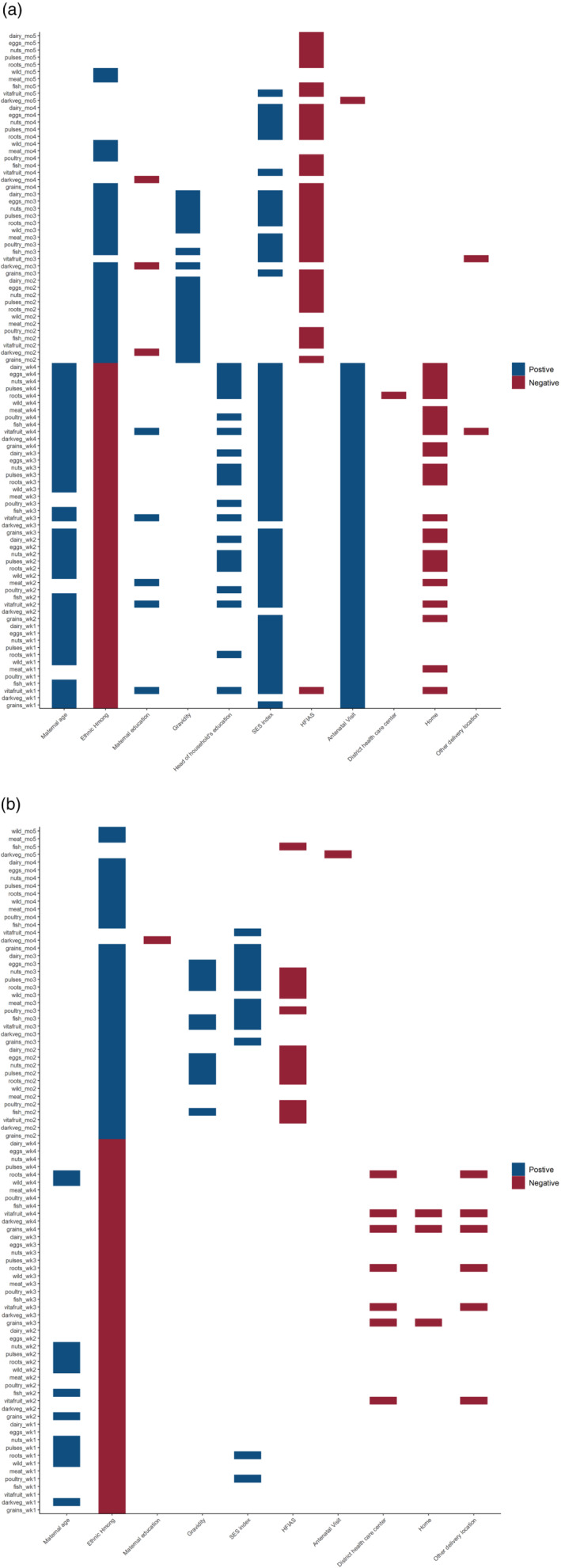
Bivariate (a) and multivariable (b) associations between maternal and household socio‐demographic characteristics and allowing food groups postpartum in weekly (for the first 4 weeks) and monthly intervals. HIFAS, household food insecurity access scale; SES index, socioeconomic status index. Positive associations indicate more likely to consume food groups and negative associations indicate less likely to consume food groups (*p* < 0.05). Predictors with *p* value <0.1 at bivariate level were included in the multivariable logistic regression model. Hmong ethnic group is compared with Lao ethnic group and district health centre; home or other delivery location is compared with delivery at a provincial hospital

In the first month postpartum in bivariate analyses (Figure 2A), factors associated with allowing more diverse food groups were older maternal age, higher number of antenatal care (ANC) visits during the pregnancy of the study child, higher maternal education (significant only for fruit), living in a household where the household head has higher levels of education and living in a household with a higher SES index (not significant for vegetables). Women belonging to the Hmong ethnic group were less likely to allow diverse food groups compared with women of Lao ethnicity, as were those who delivered their baby at home compared with a provincial hospital. In months 2 and 3 postpartum, greater gravidity, Hmong ethnic group and higher household SES index (month 3) were associated with allowing more diverse foods, while women in food insecure households were less likely to allow other grains, fruits, animal source foods, roots and tubers and eggs. In the fourth and fifth months postpartum, the associations with household SES index and food insecurity remained. There were no significant associations in month 6 or beyond or with child's sex at any postpartum time point.

In the multivariable analysis (Figure 2b), only the associations with maternal age, Hmong ethnic group and delivery location remained for month 1 postpartum, while associations with gravidity, household SES index, food insecurity and Hmong ethnic group remained in postpartum months 2 and 3 and for SES index in month 4. There were few significant associations from month 5 onwards or with maternal and household head education and ANC attendance at any timepoint.

At the food item level, bivariate and multivariable associations were similar to those seen at the food group level, with the exception of white rice and chicken. Specifically, women belonging to the Hmong ethnic group were more likely to allow white rice (as opposed to sticky rice) and chicken in the first month postpartum compared with Lao women (Table S3).

Women belonging to the Hmong ethnic group were less likely to be in the most restrictive cluster (OR: 0.19, 95% CI [0.09, 0.44]) and the least restrictive cluster (OR: 0.02, 95% CI [0.01, 0.08]) compared with the 1 month restrictive cluster. Women with greater gravidity were less likely to be in the most restrictive cluster compared with the 1 month restrictive cluster (OR: 0.80, 95% CI [0.65, 0.99]). Number of ANC visits and household food insecurity and SES index were not associated with any of the taboo diet clusters.

### 3.5 Postpartum restrictive diets and MDD‐W

**Figure 3:**
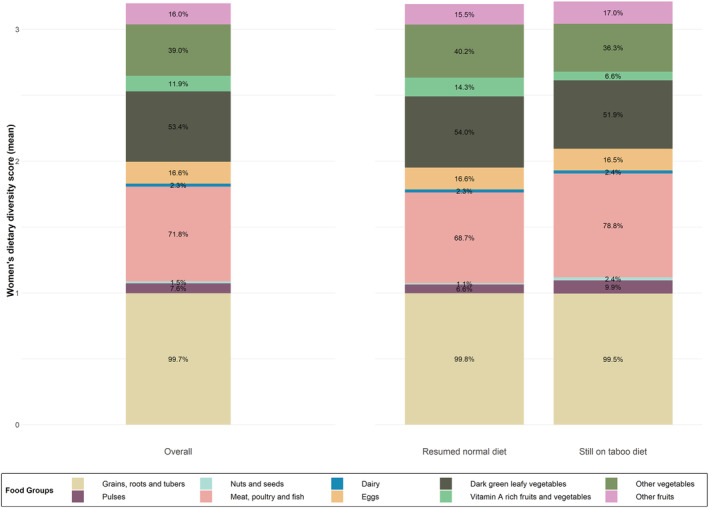
Mean dietary diversity score and percentage of women who consumed food groups in the previous 24 h/day before going to hospital, for all women (*n* = 682) and by those who were following a restrictive diet at the time of interview (*n* = 212) and those who had resumed/were consuming their normal diet (*n* = 470)

Overall, the mean ± SD dietary diversity score was 3.2 ± 1.4, and only 15% of all women achieved MDD‐W. Women in the community cohort had a higher dietary diversity score and a greater proportion achieved MDD‐W compared with women in the hospital cohort (Table 1). The proportion of women who consumed each food group in the previous 24 h/day before going to hospital is shown in Figure 3.

Of women following a taboo diet at the time of interview (*n* = 212), 10% achieved MDD‐W, compared with 17% of women who had resumed/were consuming their normal diet (*n* = 470; *p* = 0.04). The proportion of women consuming each food group was the same or similar between women still following a taboo diet compared with those who had resumed/were following their normal diet, except for meat, fish and poultry which was consumed by more women still following a taboo diet (78.8% and 68.7%, respectively) and vitamin A rich fruits and vegetables which was consumed by 14.3% of women eating their normal diet and 6.6% of women who were still adhering to a taboo diet (Figure 3).

Among women following a taboo diet at the time of the interview, those in the least restrictive cluster (OR: 6.62, 95% CI [2.74, 15.97]) and in the 1 month highly restrictive cluster (OR: 2.74, 95% CI [1.22, 6.18]) were more likely to achieve MDD‐W compared with the most restrictive cluster.

Among all women, social desirability was low (mean ± SD score 2.7 ± 1.1) and did not differ between women who met MDD‐W and those who did not (*p* = 0.76) or between those reportedly following a taboo diet at the time of interview compared with those who had resumed/were consuming their normal diet (*p* = 0.26).

## 4 DISCUSSION

Traditional postpartum dietary restrictions and food taboos were widespread among women in this study in northern Lao PDR. In contrast, restrictive diets and the concept of ‘eating down’ during pregnancy were not commonly practised. While food insecurity was associated with longer and more restrictive taboo diets, maternal age, gravidity and higher household SES were associated with allowing more diverse foods and shorter dietary restrictions. Dietary diversity was low among the majority of women and was exacerbated by culturally determined postpartum food avoidances.

It has previously been reported that 80–98% of mothers adhered to postpartum food restrictions in Lao PDR, across all regions in both rural and urban settings and different ethnic groups (Barennes et al., 2009, 2015; de Sa et al., 2013; Holmes et al., 2007). However, specific practices and taboo foods vary between and within different regions and ethnic groups. Stoeber et al. (2013) reported that postpartum food restrictions were widespread among women in Phongsaly (Northern Uplands) and Khammouane (Mekong Corridor) provinces, where women often ate only rice, galangal and salt for 15–30 days after delivery, and meat (particularly from white animals and wild animals) was avoided. In contrast, in Sekong province in the Southern Highlands, postpartum women were not dissuaded from eating any foods, with the exception of male wild animals that were considered taboo. In the present study among women in Luang Prabang and surrounding provinces, four distinct postpartum dietary patterns were identified, three of which were highly restrictive for varying lengths of time and one less restrictive cluster. Cluster analysis indicated that timing of food restrictions was better at grouping women rather than specific food groups that were avoided. White rice was universally consumed by all women at all time points, but diverse animal source foods, eggs, other grains, fruits and vegetables were restricted by the majority of women for the first 14–30 days or beyond 2 months in the most restrictive group.

The first month postpartum has been described as the most restrictive, with some women reporting that they only allow rice, galangal and salt during this time (Barennes et al., 2009; Stoeber et al., 2013). In the present study, this was especially evident among women in the Hmong ethnic group, who reportedly have some of the most restrictive postpartum cultural practices. In Hmong tradition, the first 30 days after childbirth are seen as the most dangerous period for new mothers, who are vulnerable to illness, and women must ‘confine’ themselves and are restricted in certain activities, diet and social contacts until the dangerous period is over (Rice, 2000). This includes strictly observing dietary restrictions and only eating warm chicken and rice in the first 30 days after childbirth, although some women may allow pork, fish and vegetables 10–20 days after delivery (Rice, 2000). In the present study, Hmong women reported following very restrictive diets but were more likely to allow white rice (opposed to glutinous sticky rice) and chicken in the first month postpartum compared with Lao women. Dietary restrictions and other traditional childbearing practices believed to restore health and wellbeing have been observed among Hmong women living in the United Sates (Halvorsen, 2012; Jambunathan, 1995) and urban Australia (Rice, 2000), highlighting the deeply rooted cultural beliefs among the Hmong ethnic group.

Tradition within the family or ethnic group was the most commonly cited reason for restricting all or most foods or specific foods postpartum, followed by helping the mother's body heal after childbirth, with fewer women reporting that pain or illness in the mother or infant was the main reason for restricting foods, demonstrating that there is no single reason or belief but several reasons underlying traditional food avoidances (Meyer‐Rochow, 2009). This is consistent with other studies in northern Lao PDR and Vietnam where postpartum women have reported that food taboos are passed on to them by family members, particularly their mother and mother‐in‐law, for reasons sometimes unknown (de Sa et al., 2013; Holmes et al., 2007; Lundberg & Trieu, 2011). Among women in Myanmar, well‐being of the newborn and well‐being of both the mother and the newborn were the main reasons for postpartum food avoidances (Sein, 2013). Interestingly, no women in the present study reported avoiding certain foods to improve the quality or quantity of breastmilk, in contrast to other studies in Cambodia (Wallace et al., 2014) and Vietnam (Lundberg & Trieu, 2011). Breastfeeding women in Vientiane, Lao PDR, reported drinking an unsweetened herb tea to stimulate milk production, alongside a restricted postpartum diet (Lee et al., 2013). Concerns have been raised that postpartum food restrictions may form a barrier to continued breastfeeding as women adhere to restricted diets as long as they are breastfeeding and may therefore choose to stop breastfeeding in order to resume eating their normal diet (World Food Programme, 2017). However, in this setting, in northern Lao PDR, all women reported the reason for resuming their usual diet was that it was the right time in their family/ethnic group to finish the taboo diet, and none reported breastfeeding as a reason.

Women from poorer and food insecure households reported following more restrictive taboo diets for longer periods of time, as indicated by the associations between household SES index and food insecurity in months 2–4 postpartum. It is recognized that socioeconomic factors and food insecurity are underlying causes of inadequate dietary intakes and undernutrition in low‐ and middle‐income countries (Black et al., 2008); however, this analysis also suggests an economic dimension to culturally determined postpartum taboo diets. Restrictive postpartum diets were also related to maternal age and gravidity, suggesting that women may follow less restrictive and/or shorter taboo diets following the delivery of each subsequent child, as previously noted (Barennes et al., 2009). Engagement with health care facilities and the quality of counselling provided may play a role in how women adhere to postpartum dietary restrictions. In rural Luang Prabang, Sirivong et al. (2003) found that women who lived closer to community hospitals were more likely to participate in ANC and disregard postpartum food taboos (Sirivong et al., 2003). Similarly, in urban Vientiane, multiparous women, those who attended ANC and those who delivered at hospital were less likely to adhere to dietary restrictions postpartum (Barennes et al., 2009). Nutrition interventions and counselling should be informed by knowledge of local beliefs and influencers of these beliefs, and training of health providers should include information and practical examples of how to integrate these beliefs into maternal dietary recommendations (Kavle et al., 2019; Kavle & Landry, 2018). Furthermore, community‐based participatory nutrition and behaviour change interventions, co‐designed and implemented by women, their extended family and key influencers within the community may empower women to utilize health services and adopt improved dietary practices during the perinatal period, including consumption of foods which are considered a cultural taboo (Lassi et al., 2019; Li et al., 2007). These can be implemented alongside other public health approaches to improve maternal micronutrient intakes such as food fortification and supplementation. It is important to note that factors such as SES, food insecurity, dietary practices, ANC attendance and delivery location can be related to the mothers' ethnic group, which may be driving some of the observed associations. Interestingly, in southern India, postpartum dietary restrictions were followed for 3 months following the birth of male infants but only 1 month for female infants (Sastry et al., 2021). No association with infant sex was found in the present study.

Consumption of meat, poultry and fish was higher in the previous 24 h/day before going to the hospital among women following a taboo diet at the time of the interview compared with those who had already resumed or were eating their normal diet. While this may mean women are not adhering to the traditional food avoidances indicated, the low‐to‐medium social desirability scores suggest women were not adjusting their responses to conform to social norms. Conversely, this greater consumption of meat, poultry and fish may reflect the importance of these foods postpartum and the perceived benefits to the mother. As mentioned above, chicken is an important food that is consumed as part of the Hmong postpartum diet in the 30‐day confinement period. Additionally, among the Akha ethnic group in Phongsaly province in northern Lao PDR, husbands reportedly prepared soups made from chicken and duck for their wives, which was believed to stimulate breastmilk production if the mother thought she did not have enough breastmilk (Stoeber et al., 2013). Furthermore, women who were still following a taboo diet at the time of the interview were in the latter and therefore less restrictive phases of the taboo diet, and thus, more women may have been allowing meat, poultry and fish.

Dietary restrictions or the practice of ‘eating down’ or eating less during pregnancy was not common in northern Lao PDR, with 1.6% and 11% of women reporting adherence to taboo diets or eating less food during their pregnancy, respectively. Half of women reported eating more during pregnancy. Eating down during pregnancy has been reported among women in South Asia, including India (Hutter, 1996; Mukhopadhyay & Sarkar, 2009), Bangladesh (Harding et al., 2017; Shannon et al., 2008), Pakistan (Mahmood et al., 1997) and Nepal (Christian et al., 2006), and in Southeast Asia in Myanmar (Hashmi et al., 2018) and Indonesia (Hartini et al., 2005). However, in a 2011/2012 study of pregnant women in Bangladesh, the prevalence of eating down was lower than in previous reports in similar contexts, suggesting a generational shift in this behaviour (Harding et al., 2017). Few previous studies have reported eating down in pregnancy in Lao PDR as a means of having a smaller baby and an easier delivery, although none have reported the prevalence of this practice (Holmes et al., 2007; Phimmasone et al., 1996). Hence, it is unclear if a generational shift may partly explain the low prevalence of this practice among women in northern Lao PDR.

A limitation of this analysis is the condition that for the food group to be considered allowed, ≥2 food items from that food group had to be specified as allowed (Hess et al., 2019). For example, if chicken was the only food item specified as allowed in the poultry group, it was assumed that all other types of poultry were restricted and, hence, the whole poultry food group was considered restricted in the cluster analysis. This may therefore make the cluster groups look more restrictive if women were only allowing one food item within the food group. However, associations between food items and maternal and household characteristics were identified, such as the finding that chicken was more likely to be allowed by women of Hmong ethnicity compared with those of Lao ethnicity. No data were collected on dietary intakes of mothers, and it is not known how often women actually consume any of the reported allowed foods. For example, among women in Luang Namtha province, northern Lao PDR, women adhering to postpartum dietary restrictions reported that they ate chicken and eggs three times per week, or chicken only twice a month (Barennes et al., 2015). Finally, this study was conducted in Luang Prabang and surrounding provinces in northern Lao PDR where Lao, Khmu and Hmong are the predominant ethnic groups. The study population participated in a prospective cohort study and is neither representative of these provinces, nor are these findings generalizable to other regions of Lao PDR or other ethnic groups. Qualitative analysis would have allowed for further in‐depth exploration of the underlying socio‐cultural beliefs surrounding food avoidances among these women; however, strengths of this study included the large sample size and the in‐depth taboo diet questionnaire that determined which food groups and food items were either allowed or restricted by women in weekly intervals for the first month postpartum when diets are the most restrictive, and monthly intervals thereafter. This allowed for novel cluster analysis that identified distinct dietary patterns among women following postpartum food restrictions.

## 5 CONCLUSION

Culturally determined restrictive diets and food avoidances are common among postpartum women in Lao PDR. These highly restrictive diets combined with low dietary diversity and household food insecurity likely contribute to micronutrient deficiencies in mothers that may have important consequences for their breastfed infants through reduced micronutrient content of breastmilk, which requires further exploration. As perinatal food avoidances are deeply rooted cultural traditions often passed through generations, several interventions may be required to improve dietary and micronutrient intakes among women. Culturally appropriate strategies to increase micronutrient intakes among women, such as multiple micronutrient supplementation, food fortification and community‐based participatory nutrition and behaviour change interventions, should be considered.

## CONFLICT OF INTEREST

The spouse of SYH previously worked for the Bill & Melinda Gates Foundation. All other authors declare that they have no conflict of interest.

## CONTRIBUTIONS

SYH conceived and designed the study protocol; TJS and SYH developed the data collection questionnaires; SK translated all data collection questionnaires to Lao language; XT programmed all data collection questionnaires; TJS, DS, SK and SYH planned the local study implementation; TJS and DS supervised the data collection; TJS, XT, CDA and SYH analysed and interpreted the data; TJS wrote the manuscript. All authors critically reviewed the manuscript and read and approved the final version.

## TRIAL REGISTRATION

This trial was registered at clinicaltrials.gov as NCT03626337.

## Supporting information


**Table S1.** Percent of women within each cluster who reported that they would allow food groups in months 1 and 2 postpartum
**Table S2.** Proportion of women of each ethnic group in the postpartum taboo diet clusters
**Table S3.** Associations between allowing white rice and chicken in the first month postpartum among women belonging to the Hmong ethnic group compared to women of Lao ethnicity
**Figure S1.** Flowchart of mother's eligibility, enrollment and data collection in the hospital and community cohorts
**Figure S2.** Proportion of women within each ethnic group who reported allowing food groups during pregnancy and postpartum

## Data Availability

The data that support the findings of this study are openly available online (at https://osf.io/jfke3/)
