# Supplementary material for: Traditional prenatal and postpartum food restrictions among women in northern Lao PDR
Source: Matern Child Nutr. 2021 Sep 30;18(1):e13273. doi: 10.1111/mcn.13273 (PMC8710103; doi:10.1111/mcn.13273)
Supplement: Supplementary file 1 — Table S1. Percent of women within each cluster who reported that they would allow food groups in months 1 and 2 postpartum Table S2. Proportion of women of each ethnic group in the postpartum taboo diet clusters Table S3. Associations between allowing white rice and chicken in the first month postpartum among women belonging to the Hmong ethnic group compared to women of Lao ethnicity Figure S1. Flowchart of mother's eligibility, enrollment and data collection in the hospital and community cohorts Figure S2. Proportion of women within each ethnic group who reported allowing food groups during pregnancy and postpartum [file MCN-18-e13273-s001.pdf]

Table S1. Percent of women within each cluster who reported that they would allow food groups in months 1 and 2 postpartum

Food group	Timepoint	Most restrictive (n = 127)	Least restrictive (n = 99)	2 weeks restrictive (n = 31)	1 month restrictive (n = 415)
**Other grains**[†]	Week 1	3	80	0	0
	Week 2	3	100	3	0
	Week 3	2	100	61	0
	Week 4	2	100	100	0
	Month 2	2	100	100	100
**Meat**	Week 1	0	70	0	0
	Week 2	0	84	3	0
	Week 3	0	84	55	0
	Week 4	0	84	94	0
	Month 2	0	87	94	98
**Poultry**	Week 1	2	78	3	0
	Week 2	2	99	6	0
	Week 3	2	99	65	0
	Week 4	2	99	100	0
	Month 2	2	99	100	100
**Fish**	Week 1	3	79	0	2
	Week 2	5	98	0	2
	Week 3	4	98	58	2
	Week 4	4	98	97	2
	Month 2	4	98	97	100
**Wild animals**	Week 1	2	72	0	0
	Week 2	2	92	0	0
	Week 3	1	92	52	0
	Week 4	1	92	90	0
	Month 2	1	94	90	98
**Eggs**	Week 1	4	80	10	1
	Week 2	4	100	13	1
	Week 3	3	100	68	1
	Week 4	3	100	100	1
	Month 2	3	100	100	100
**Vegetables**	Week 1	21	84	13	12
	Week 2	22	100	19	13
	Week 3	23	100	68	13
	Week 4	25	100	100	13
	Month 2	26	100	100	100
**Fruits**	Week 1	9	80	0	2
	Week 2	9	100	6	2
	Week 3	8	100	58	2
	Week 4	9	100	97	2
	Month 2	10	100	97	100
**Roots and tubers**	Week 1	0	80	0	0
	Week 2	0	100	3	0
	Week 3	0	100	61	0
	Week 4	0	100	100	0
	Month 2	0	100	100	100
**Pulses**	Week 1	0	80	0	0
	Week 2	0	100	3	0
	Week 3	0	100	61	0
	Week 4	0	100	100	0
	Month 2	0	100	100	100

**Nuts and seeds**	Week 1	0	80	0	0
	Week 2	0	100	3	0
	Week 3	0	100	61	0
	Week 4	0	100	100	0
	Month 2	0	100	100	100
**Dairy**	Week 1	2	80	0	0
	Week 2	2	100	3	0
	Week 3	1	100	61	0
	Week 4	1	100	100	0
	Month 2	1	100	100	100

† Excludes rice

Table S2. Proportion of women of each ethnic group in the postpartum taboo diet clusters

Taboo diet cluster	Lao (n = 102)	Khmu (217)	Hmong (n = 328)	Other (n = 25)
**Most restrictive**	20 (20)	65 (30)	33 (10)	9 (36)
**Least restrictive**	32 (31)	56 (26)	5 (2)	6 (24)
**2 weeks restrictive**	6 (6)	13 (6)	11 (3)	1 (4)
**1 month restrictive**	44 (43)	83 (38)	279 (85)	9 (36)

Values represent n (%)

Table S3. Associations between allowing white rice and chicken in the first month postpartum among women belonging to the Hmong ethnic group compared to women of Lao ethnicity

Postpartum time point	OR (95% CI)[a]		OR (95% CI)[b]	
	White rice	Chicken	White rice	Chicken
**Week 1**	19.2 (9.0, 40.8)	17.7 (9.0, 34.7)	37.9 (15.4, 93.6)	18.4 (8.4, 40.0)
**Week 2**	16.6 (7.8, 35.6)	11.2 (5.7, 21.9)	31.1 (12.6, 76.6)	11.4 (5.3, 24.4)
**Week 3**	15.66 (7.3, 33.7)	10.6 (5.3, 21.2)	28.9 (11.7, 71.5)	9.8 (4.6, 20.8)
**Week 4**	15.9 (7.4, 34.2)	8.9 (4.4, 17.9)	28.4 (11.4, 70.7)	8.4 (3.9, 17.8)

[a] Bivariate associations
[b] Multivariable associations
p value <0.001 for all associations shown

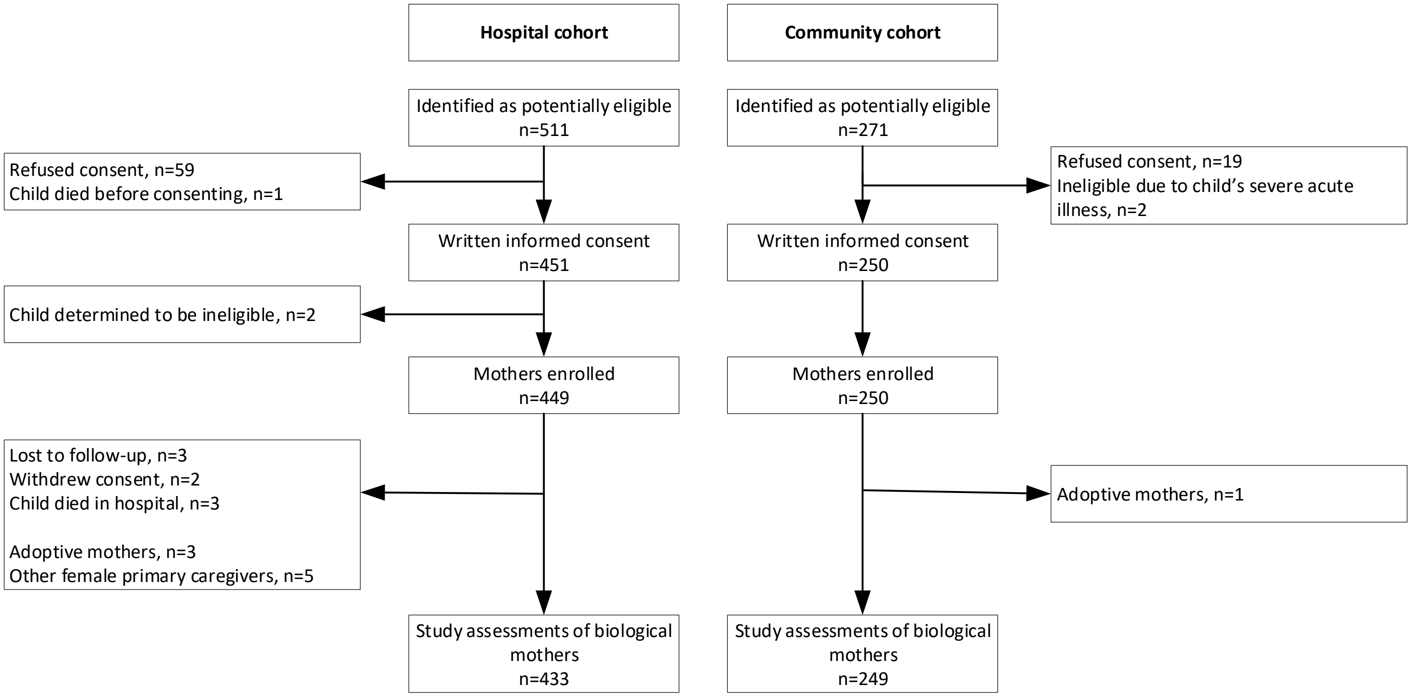

Figure S1. Flowchart of mother's eligibility, enrollment and data collection in the hospital and community cohorts

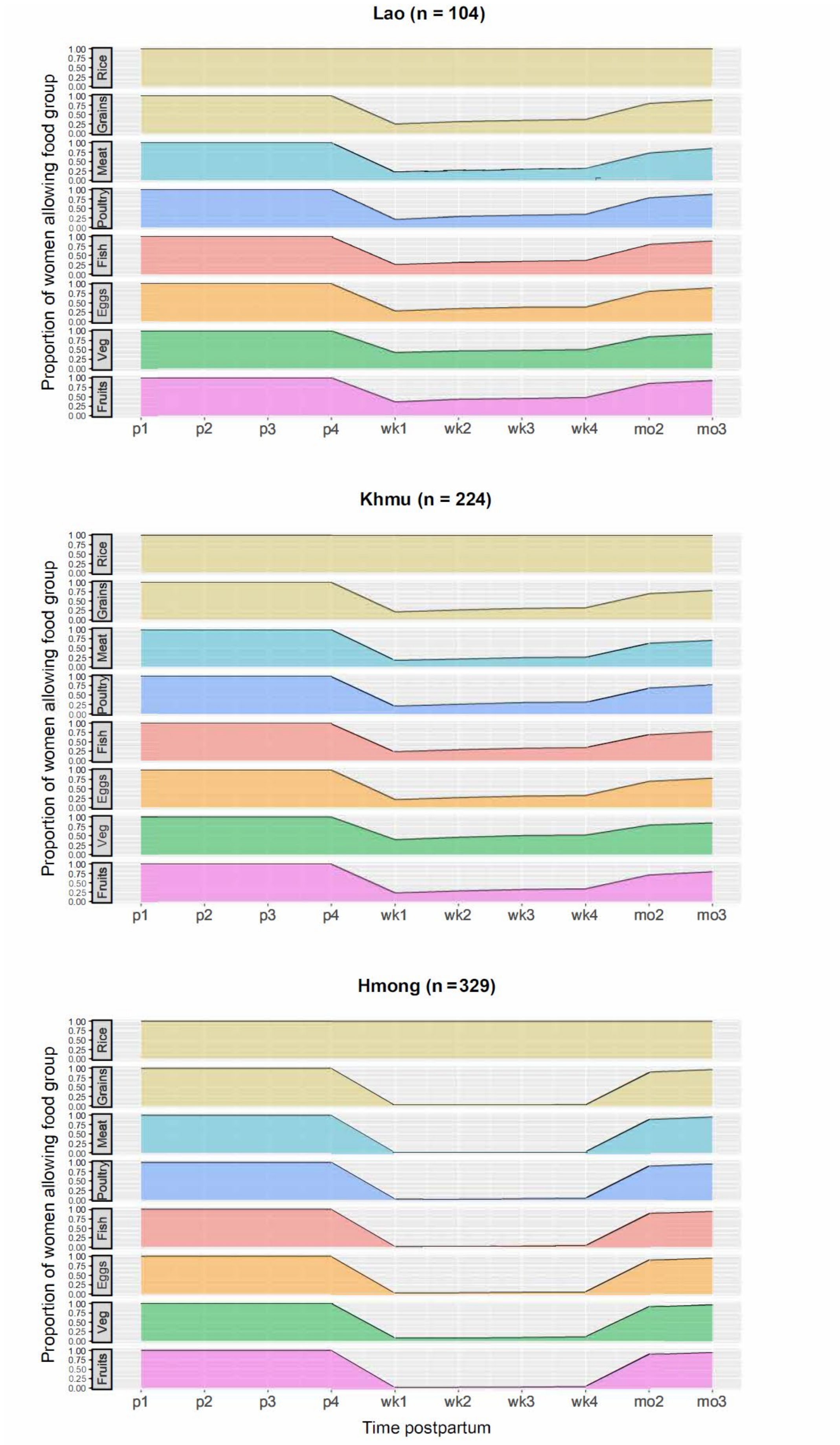

Figure S2. Proportion of women within each ethnic group who reported allowing food groups during pregnancy and postpartum

p1 = trimester 1; p2 = trimester 2; p3 = trimester 3; p4 = before birth; postpartum is in weekly for the first 4 weeks (wk1 – wk4) and monthly intervals thereafter (mo2 – mo3)
